# PFKFB3 Regulates Chemoresistance, Metastasis and Stemness *via* IAP Proteins and the NF-κB Signaling Pathway in Ovarian Cancer

**DOI:** 10.3389/fonc.2022.748403

**Published:** 2022-01-28

**Authors:** Yu-xin Jiang, Michelle K. Y. Siu, Jing-jing Wang, Thomas H. Y. Leung, David W. Chan, Annie N. Y. Cheung, Hextan Y. S. Ngan, Karen K. L. Chan

**Affiliations:** ^1^ Department of Obstetrics and Gynaecology, University of Hong Kong, Hong Kong, Hong Kong SAR, China; ^2^ Department of Gynaecology, The First Affiliated Hospital of Nanjing Medical University, Jiangsu, China; ^3^ Department of Pathology, University of Hong Kong, Hong Kong, Hong Kong SAR, China

**Keywords:** PFKFB3, stemness, PFK158, therapeutic target, ovarian cancer

## Abstract

Glycolysis has been reported to be critical for cancer stem cells (CSCs), which are associated with tumor chemoresistance, metastasis and recurrence. Thus, selectively targeting glycolytic enzymes may be a potential therapy for ovarian cancer. 6‐phosphofructo‐2‐kinase/fructose‐2,6‐biphosphatase 3 (PFKFB3), the main source of fructose-2,6-bisphosphate, controls the first committed step in glycolysis. We investigate the clinical significance and roles of PFKFB3 in ovarian cancer using *in vitro* and *in vivo* experiments. We demonstrate that PFKFB3 is widely overexpressed in ovarian cancer and correlates with advanced stage/grade and poor outcomes. Significant up-regulation of PFKFB3 was found in ascites and metastatic foci, as well as CSC-enriched tumorspheres and ALDH+CD44+ cells. 3PO, a PFKFB3 inhibitor, reduced lactate level and sensitized A2780CP cells to cisplatin treatment, along with the modulation of inhibitors of apoptosis proteins (c-IAP1, c-IAP2 and survivin) and an immune modulator CD70. Blockade of PFKFB3 by siRNA approach in the CSC-enriched subset led to decreases in glycolysis and CSC properties, and activation of the NF-κB cascade. PFK158, another potent inhibitor of PFKFB3, impaired the stemness of ALDH+CD44+ cells *in vitro* and *in vivo*, whereas ectopic expression of PFKFB3 had the opposite results. Overall, PFKFB3 was found to mediate metabolic reprogramming, chemoresistance, metastasis and stemness in ovarian cancer, possibly *via* the modulation of inhibitors of apoptosis proteins and the NF-κB signaling pathway; thus, suggesting that PFKFB3 may be a potential therapeutic target for ovarian cancer.

## Introduction

Ovarian cancer is the leading cause of death in gynecologic malignancies with 75% of patients presenting late at the advanced stages. Optimal treatment involves primary cytoreductive surgery followed by adjuvant chemotherapy with cisplatin. Unfortunately, the prognosis is not optimistic due to chemoresistance and frequent relapse. Identifying the related molecular mechanisms can help to develop alternative therapeutic strategies for ovarian cancer (1). Unlike other tumors, ovarian cancer has a unique pattern of metastasis. Because of the lack of an anatomical barrier, ovarian cancer cells are likely to separate from the primary lesion, shed and flow into the abdominal cavity and spread *via* the peritoneal fluid which allows cancer cells to spread into the distant organs (2). Most advanced ovarian cancer patients are diagnosed with accumulation of peritoneal fluid (also known as ascites fluid). Previous studies have indicated that “spheroids” or “tumorspheres” can be enriched in ascites from patients with ovarian cancer with enhanced tumorigenesis and cancer stem cell (CSC) properties (3, 4). Accumulating evidence suggests that CSCs or tumor-initiating cells may be the source of all tumor cells and a cause of metastasis, chemoresistance and recurrence (5). Thus, targeting CSCs may be a novel therapeutic strategy for ovarian cancer.

Accumulating evidence suggests that glycolysis is critical for CSC maintenance (6). Pyruvate dehydrogenase (PDH), a key enzyme responsible for switching pyruvate toward the tricarboxylic acid cycle in mitochondria and away from glycolysis, is diminished in CD44+/MyD88+ ovarian CSCs (7). A subset of radio-resistant ovarian cancer cells has been identified with CSC-like aggressive properties, up-regulated glucose transporter 1 (GLUT1) expression and an elevated extracellular acidification rate. Moreover, 188Re-Liposome, an anti-cancer drug, has been found to suppress tumor growth by shifting glycolysis toward oxidative phosphorylation (OXPHOS) (8). With metabolic assays, spheroids have been derived from ascites with enhanced glycolytic influx and resistance to oxygen deprivation (3). These studies have indicated the crucial role of glycolysis in ovarian CSCs regulation, although the underlying mechanism remains unknown.

Phosphofructokinase 1 (PFK1), a key enzyme in glycolysis, is regulated by fructose-2,6-bisphosphate (F-2,6-BP). F-2,6-BP is induced by 6‐phosphofructo‐2‐kinase/fructose‐2,6‐biphosphatase 3 (PFKFB3) *via* its kinase activity, thereby enhancing glycolysis. PFKFB3 has been reported to be up-regulated in malignant tumors compared with normal tissue in leukemia and several human cancers, including colorectal, breast, lung, thyroid and nasopharyngeal cancer (9–13). Up-regulation of PFKFB3 expression in nasopharyngeal (12) and breast (13) cancers promotes tumor cell proliferation, metastasis and angiogenesis. Beyond glycolysis, PFKFB3 has been revealed to be involved in the cell cycle in breast cancer cells by degrading p27, which inhibits G1/S transition and promotes apoptosis (14). A recent study has suggested that high expression of PFKFB3 indicates poor prognosis and enhances the self-renewal capacity of CSCs by *in vivo* extreme limiting dilution assays in breast cancer (15). However, the role of PFKFB3 in tumorigenesis, CSCs regulation of ovarian cancer, remains to be further elucidated.

Firstly, the clinical significance of PFKFB3 in ovarian cancer was assessed. We then investigated the effects and mechanism of PFKFB3 on ovarian cancer metabolic switch and regulation of chemoresistance to cisplatin using one of its inhibitors 3PO. In our recent study, we found that ALDH+CD44+ cells isolated from ascites-derived tumor cells show enhanced CSC properties (16). Here, we isolated tumorspheres or an ALDH+CD44+ cell subset from ascites or ascites-originating ovarian cancer cell lines (SKOV3 and OVCAR3) and compared the PFKFB3 expression between the CSC-enriched subpopulation and non-CSCs. Functional assays were conducted, and the downstream signaling pathway through which PFKFB3 affects stemness in the CSC-enriched subpopulation was studied using siRNA approach and treatment with another PFKFB3 inhibitor, PFK 158. Our *in vitro* and *in vivo* results show that PFKFB3 was overexpressed in ovarian cancer, ascites-derived tumor cells and CSC-enriched subpopulations. Knockdown of PFKFB3 or blocking PFKFB3 with 3PO/PFK158 reduced lactate production, sensitized resistant cells to cisplatin treatment and inhibited CSC properties and tumor growth. In contrast, overexpression of PFKFB3 led to the opposite effects. The mechanisms involve inhibition of apoptosis and activation of NF-κB signaling pathway. Thus, PFKFB3 might be used as a potential therapeutic target in treating ovarian cancer.

## Materials and Methods

### Clinical Samples and Cell Lines

**Figure 1:**
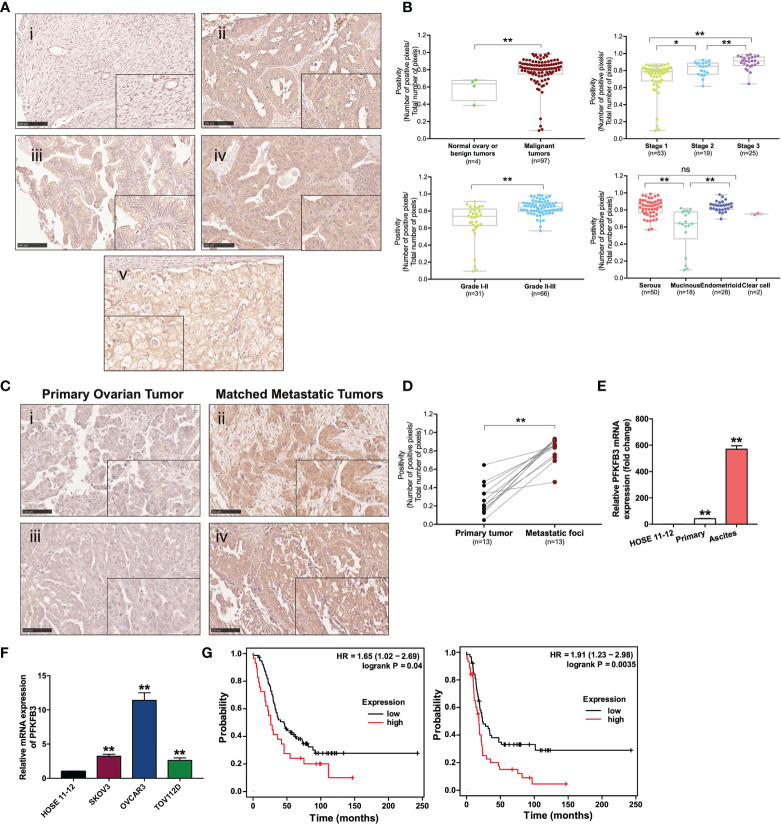
Overexpression of PFKFB3 in ovarian cancer is correlated with metastasis and poor prognosis. **(A)** Representative images of PFKFB3 expression in (i) normal ovarian tissue, (ii) serous, (iii) mucinous, (iv) endometrioid and (v) clear cell subtypes of tumor samples on ovarian cancer TMA (OVC1021, Biomax) by IHC staining (scale bar, 100 μm). The insets highlight regions with higher magnification. **(B)** Association of PFKFB3 with stage/grade/histotype in ovarian cancer. The graphs for stage and grade encompass all four histotypes. **(C)** Representative images of IHC staining for PFKFB3 expression in ovarian primary tumors (i, iii) and their metastatic foci (ii, iv; scale bar, 100 μm). **(D)** Bar chart showing PFKFB3 staining in 13 pairs of tissues. **(E)** Relative PFKFB3 mRNA expression in HOSE 11-12, primary cancer cells and matched ascites cancer cells from high-grade serous ovarian cancer, determined by qPCR. **(F)** PFKFB3 expression in HOSE 11-12 and ovarian cancer cell lines (SKOV3, OVCAR3, and TOV112D) at the mRNA level, determined by qPCR. **(G)** (left) Overall and (right) progression-free survival rates were analyzed with the log-rank test for PFKFB3 high/low groups of ovarian cancer patients obtained from GSE26193 (n = 107), by using the Kaplan-Meier plotter. (*p < 0.05, **p < 0.01; n.s., not significant).

A tissue microarray analysis (TMA, OVC1021; Biomax) with 102 cores from four normal benign and 97 ovarian cancer tissues was performed in duplicate. Number of samples in each categories were shown in **Figure 1B**. Clinicopathological characteristics of 97 samples were shown in **Supplementary Table S1**. Archived formalin-fixed, paraffin-embedded (FFPE) samples from 13 pairs of advanced-stage high-grade serous primary ovarian cancer tissues and their matched metastatic foci from the Queen Mary Hospital (University of Hong Kong) were used for this study. Fresh ascitic fluid and primary tissue samples were obtained from patients underwent tumor-debulking surgery for serous, clear cell and endometrioid ovarian cancer, followed by chemotherapy. Primary cancer cells and matched ascites cancer cells were from serous ovarian cancer. Tumorsphere/monolayer cancer cells derived from ascites were from serous and clear cell ovarian cancers. Ascites cancer cells used for functional assays after transient knockdown of PFKFB3 were from serous and endometrioid ovarian cancers. Ascitic fluid and tissue samples were isolated into single cells. Briefly, solid tumor tissues were finely minced with scissors, incubated with Ca2**^+^**/Mg2**^+^**-free PBS containing 1 mg/mL collagenase/dispase (Roche, UK) at 37°C for 80 min, filtered through a sterile cell strainer (40 μm; Corning, NY, USA), and centrifuged at 100 g for 10 min. The ascitic fluid was centrifuged at 100 g for 10 min, the supernatant fraction was discarded, and the cell pellet was collected. To exclude erythrocytes, we purified the obtained single cells with various solutions of NaCl. The isolated cells were cultured in 1:1 mixture of MCDB 105 medium and Medium 199 (Sigma-Aldrich) with 10% fetal bovine serum (Gibco) and 1% penicillin-streptomycin. The use of patient samples was approved by the Institution Ethical Review Board of the University of Hong Kong (UW 16-107). The normal human ovarian surface epithelial cell line HOSE 11-12 was provided by Professor SW Tsao (School of Biomedical Sciences, University of Hong Kong). Three human ovarian cancer cell lines, SKOV3, OVCAR3 and TOV112D, were purchased from ATCC (Manassas, VA, USA). A2780S, a cisplatin chemosensitive cell line, and its chemoresistant counterpart A2780CP, were provided by Prof. Benjamin B.K. Tsang (Department of Obstetrics & Gynaecology and Cellular & Molecular Medicine, University of Ottawa).

### Tumorsphere Culture

Single cells were seeded into six-well ultra-low attachment plates at a density of 2000–5000 cells/well containing serum-free DMEM/F12 (1:1) (Gibco, MD, USA) supplemented with 10 ng/mL basic fibroblast growth factor (Sigma-Aldrich, MO, USA), 20 ng/mL human epidermal growth factor (Sigma-Aldrich) and 5 μg/mL insulin. Every 2 days, fresh medium was added to each well without removal of the old medium. Tumorspheres, defined as multicellular cell aggregates floating in the medium 7-14 days after culture, were used for mRNA and protein extraction, as well as for functional assays.

### Fluorescence-Activated Cell Sorting

Fluorescence-activated cell sorting (FACS) was used to isolate the ALDH and CD44 double-positive/double-negative subpopulation. Briefly, cells were suspended and incubated in ALDEFLUOR™ Assay Buffer containing ALDEFLUOR™ reagent (StemCell Technologies, Canada) at 37°C for 30 min and centrifuged for 10 min at 300 g; the supernatant was then discarded. APC-conjugated CD44 antibody (Miltenyi Biotec, Germany) was added to the cells resuspended in Flow Buffer (Miltenyi Biotec, Germany) with the FcR Blocking Reagent. Then the mixture was incubated in the dark for 30 min at 4°C. Cells were stained with the ALDH inhibitor diethylaminobenzaldehyde (DEAB), and anti-IgG-APC was used as a negative control for flow cytometry gating.

### Transient Transfection of PFKFB3

For transient knockdown, two commercially obtained PFKFB3 siRNA (Cat#: 4427038; siRNA #1 ID:s10357; siRNA #2 ID:s10359; Ambion; USA) (transfected as duplexes) and control siRNA (Cat#: 4390846; Ambion; USA) were introduced into cancer cells *via* SilentFect reagent (Bio-Rad, Canada). For transient overexpression, PFKFB3 (Cat#: RC22748L2) and control vector (Cat#: PS100071) plasmids (GFP-tagged; OriGene, MD, USA) were introduced into cancer cells with Lipofectamine 3000 reagent (Invitrogen, Canada). For transfection in tumorspheres, spheres were dissociated into single cells and seeded into six-well ultra-low attachment plates 24 h before transfection (17). The cells were harvested and plated for the subsequent functional assays 48 h after transfection.

### Drug Treatment

For cisplatin treatment, A2780S and A2780CP cells were plated 24 h before treatment with cisplatin (0, 2.5, 5 and 10 µM) (Invitrogen, Canada) for 48 h. For 3PO and cisplatin co-treatment, A2780CP cells were plated 24 h before treatment with cisplatin (20 µM) and 3PO (10 µM) (Sigma, USA). ddH2O and DMSO were used as vehicle for cisplatin and 3PO, respectively. For PFK158 treatments, ALDH+CD44+ SKOV3 cells were plated 12 h before treatment with PFK158 (0.5, and 1.0 μM) (Axon Medchem, Holland) or control vehicle (ddH2O) for 48 h.

### Immunohistochemistry

Immunohistochemistry (IHC) analysis of TMA and FFPE-tissue sections was performed according to the manufacturer’s instructions. Briefly, the sections were incubated with anti-PFKFB3 antibody (rabbit anti-PFKFB3, 1:100; Cat. #AP8145b, ABGENT, San Diego, USA) at 4°C overnight in the dark and then incubated with Labelled Polymer-HRP solution (Dako, CA, USA) and 1% 3, 3-diaminobenzidine-hydrogen peroxide (Sigma-Aldrich). The expression was analyzed with a positive pixel count algorithm by ImageScope software (www.aperio.com). According to positive and negative control tissues. the color saturation threshold, with the peak (1.0) and bottom (0) limits for the strongest and weakest pixel settings was set by the software. Based on the intensity and quantity of positive pixels, which was normalized to the number of pixels counted in the selected representative areas, positive pixel counting (positivity) was calculated automatically. Five representative areas were selected and an average score obtained for each core of TMA and sample tissue section. Negative control with primary antibody replaced with PBS was performed.

### Real-Time PCR

Total RNA was extracted from ovarian cancer cells with a NucleoSpin^®^ RNA Kit (Macherey-Nagel, Germany) and used to generate cDNA with SuperScript VILO™ Master Mix (Invitrogen) according to the manufacturer’s instructions. The expression levels of mRNAs were quantified with the ABI Prism 7700 platform (Life Technologies) with SYBR Green qPCR Master Mix (ExCell, China). The relative mRNA expression levels were assessed with the comparative Ct method with GAPDH as the reference gene. The primers used in this study are as follows: GAPDH forward 5´ to 3´ TCCATGACAACTTTGGTATCGTG, reverse 5´ to 3´ ACAGTCTTCTGGGTGGCAGTG; PFKFB3 forward 5´ to 3´ CAGTTGTGGCCTCCAATATC, reverse 5´ to 3´ GCTTCATAGCAACTGATCC; KLF4 forward 5´ to 3´ GGGAGAAGACACTGCGTCAA, and reverse 5´ to 3´ GGAAGTCGCTTCATGTGGGA.

### Human Apoptosis RT^2^ Profiler PCR Array

To examine the apoptotic pathways by which 3PO inhibits the chemoresistant phenotype of cancer cells, the expression of 84 apoptosis-related genes in cisplatin (20 µM) and 3PO (10 µM) treatment was evaluated using the human apoptosis RT^2^ profiler PCR array (PAHS-012Z, Qiagen, Germany). qPCR was performed on a real-time PCR System (ABI Vii7 Fast, Life Technologies). Data analysis was performed by the RT^2^ profiler PCR array software with normalization genes automatically selected from the full plate.

### Immunoblot Analysis

Protein was prepared by lysing cells in CelLyticTM M solution (Sigma-Aldrich) with a protease inhibitor cocktail. After protein quantification with a NanoDrop 2000/2000c spectrophotometer (Thermo Scientific, US), equal amounts of protein lysates were loaded and resolved by SDS-PAGE and transferred to a polyvinylidene difluoride membrane. Protein signals were immunodetected with the appropriate antibodies (rabbit anti-PFKFB3, 1:1000, ABGENT, AP8145b; rabbit anti-PARP, 1:1000, CST, #9542; rabbit anti-c-IAP1, 1:1000, CST #7065; rabbit anti-c-IAP2, 1:1000, CST #3130; and rabbit anti-survivin, 1:1000, CST #2808; PARP, 1:1000, CST, #9542; rabbit anti-KLF4, 1:1000, CST, #4038; rabbit anti-BMI1, 1:1000, CST, #58565; rabbit anti-p-p65, 1:1000, CST, #3033; rabbit anti-p65, 1:1000, CST, #4765; and mouse anti-Actin, 1:50, 000, Abcam, ab6276).

### Metabolic Assays

Lactate levels and oxygen consumption rate were measured with a Lactate Colorimetric Assay Kit II (BioVision, Canada) and Extracellular Oxygen Consumption Assay (Abcam), according to the manufacturers’ instructions and normalized to cell number.

### Annexin-V-FLUOS Staining

The apoptosis status of cells after 3PO treatment with or without cisplatin treatment was evaluated by Annexin-V-FLUOS Staining Kit (Roche) following the protocol provided by the manufacturer. Briefly, the harvested single cells were incubated with FITC-conjugated annexin-V reagent and PI and detected by flow cytometry analysis. Early apoptosis (annexin V+PI-) and late apoptosis (annexin V+PI+) were included into apoptotic cells.

### Transwell Migration and Invasion Assays

Transwell migration and invasion assays were conducted to measure cellular capacity to migrate and invade *in vitro*. For migration and invasion assays using tumorspheres, spheres were dissociated into single cells before cell plating. Briefly, an equal quantity (1.25×10^5^) of cells cultured in serum-free medium was transferred to the top chamber with an 8.0 μm pore polycarbonate membrane for migration and a Matrigel-coated membrane for invasion. The lower chamber was filled with 10% fetal bovine serum medium. The assays continued for 12–24 h, and cells that had migrated/invaded were fixed, stained with 0.5% crystal violet and imaged. The migration/invasion capacity was normalized to that of control cells.

### Clonogenic Assays

Cancer cells were serially diluted and seeded in triplicate into a six-well plate at a density of 500 cells/well and colony formation was performed over 10 to 14 days. Then, cells were stained with 0.05% crystal violet, and colonies of >50 cells were counted.

### Sphere Formation Assays

Sphere formation assays were conducted by culturing cancer cells with CSC medium in six-well ultra-low attachment plates at a density of 2000–5000 cells/well and allowing the cells to grow for 7–14 days. Then, 300 μl fresh CSC medium was added to the plates every second day. Spheres with diameters >50 μm were counted.

### *In Vivo* Tumorigenicity Analysis *via* Subcutaneous Implantation and Limiting Dilution Assays

For *in vivo* tumorigencity analysis, BALB/c nude mice (7–8 weeks) with T cell-deficiency were used, according to protocols approved by the Committee of the Use of Live Animals in Teaching and Research (CULATR) under an approved license (No. 4598-18). FACS-sorted ALDH+CD44+ SKOV3 cells were pre-treated with PFK158 (1.0 μM) or control vehicle (ddH**_2_** O) for 3 days, collected, suspended in a mixture of RPMI medium and Matrigel (High Concentration, Corning), and subcutaneously injected into the flanks of mice after serial dilution (3×10**^5^**, 1×10**^4^**, 5×10**^3^**, or 2.5×10**^3^** cells) (18). Each group consisted of five mice, and tumor incidence was recorded. The estimated frequency of CSCs was calculated *via* Extreme Limiting Dilution Analysis software (http://bioinf.wehi.edu.au/software/elda/). For the group injected with 3×10^5^ cells, tumor formation time, size and weight were recorded every 3 to 5 days for 28 days. The mice were euthanatized at 28 days post tumor inoculation with 150 mg/kg pentobarbital after the experiment.

### Cancer Genome Atlas Dataset (TCGA) and Gene Expression Profiling Interactive Analysis (GEPIA)

The online software GEPIA (www.gepia.cancer-pku.cn) was used to analyze the correlation between target genes and PFKFB3 in ovarian cancer on the basis of the TCGA database.

### Statistical Analysis

The data are shown as the means ± SEM of triplicate experiments. GraphPad Prism 7.0 software was used for data analysis with a two-tailed t-test or one-way ANOVA. The survival rate was analyzed with Kaplan-Meier Plotter (www.kmplot.com), and hazard ratios and log-rank P-values were calculated automatically on the basis of the optimal cutoff value auto-selected in the software. Differences were considered statistically significant at p < 0.05 (* represents p<0.05 and ** p<0.01).

## Results

Overexpression of PFKFB3 in ovarian cancer correlates with metastasis and poor prognosis to study the role of PFKFB3 in ovarian cancer, we analyzed two independent microarray databases from Oncomine. Statistical up-regulation of PFKFB3 was observed in ovarian cancer tissues compared with normal ovary tissues (**Table 1**). TMA analysis was used to study the expression of PFKFB3 in ovarian cancer tissues. In the cytoplasm, PFKFB3 was found to be more highly expressed in malignant tumors than in normal tissues/benign tumors (**Figure 1A**). Specifically, PFKFB3 was significantly up-regulated in higher grades and stages (**Figure 1B**). For histologic subtypes, serous and endometrioid subtypes had significantly higher expression than the mucinous subtype (**Figure 1B**). Since the sample number of clear cell subtype is small (n=2), we have not used this subtype for statistical analysis. In addition, 13 pairs of FFPE tissue sections composed of metastatic foci and matched primary ovarian tumors were examined through PFKFB3 staining with IHC (**Figure 1C**). Statistically higher expression of PFKFB3 was observed in the metastatic foci (**Figure 1D**). No staining was observed in negative control with primary antibody replaced with PBS (**Supplementary Figure 1**). PFKFB3 was further detected to be up-regulated in ascites-derived tumor cells compared with primary tumor cells (**Figure 1E**) by qPCR; thus, suggesting that PFKFB3 is associated with metastasis in ovarian cancer. Moreover, Real-time PCR (qPCR) revealed up-regulation of PFKFB3 in ovarian cancer cell lines (SKOV3, OVCAR3 and TOV112D) compared with the HOSE 11-12 cell line (**Figure 1F**).

**Table 1:** Detailed information about the 3 public expression datasets of Oncomine database about PFKFB3 in ovarian cancer.

Datasets (sample size)	Comparison Groups	Fold Change	P value	Overexpression Gene Rank
Lu Ovarian (50)	Ovarian Serous Adenocarcinoma vs. Normal	1.596	0.007	2184 (in top 13%)
	Ovarian Mucinous Adenocarcinoma vs. Normal	2.015	0.011	1224 (in top 7%)
Welsh Ovarian (32)	Ovarian Serous Surface Papillary Carcinoma vs. Normal	2.079	0.008	1478 (in top 28%)

To investigate the prognostic value of PFKFB3 in ovarian cancer at the mRNA level, we analyzed a clinical database (GSE26193) containing data from 107 patients, by using Kaplan-Meier Plotter with the auto-selected best cutoff. Higher PFKFB3 was associated with poorer overall (p=0.04) and progression-free (p=0.0035) survival; thus, suggesting a correlation between PFKFB3 and poor outcomes in ovarian cancer (**Figure 1G**).

### PFKFB3 Regulates Chemoresistance in Ovarian Cancer

**Figure 2:**
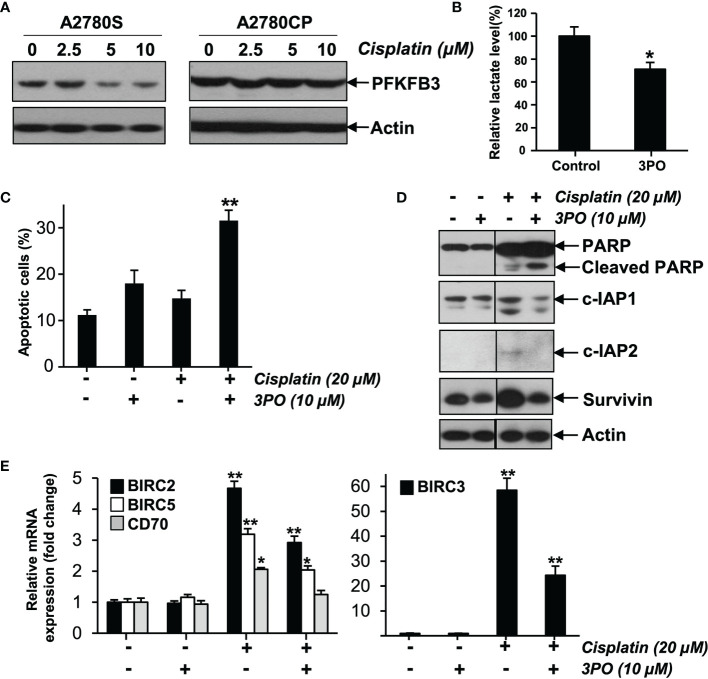
PFKFB3 regulates chemoresistance in ovarian cancer. **(A)** Protein levels of PFKFB3 in A2780S and A2780CP cells treated with cisplatin (0, 2.5, 5 and 10 µM) for 48 h, determined by immunoblotting. **(B)** Relative lactate production level of A2780CP cells treated with 3PO (0, 10 µM), determined by Lactate Colorimetric Assay Kit II. **(C)** A2780CP cells treated with 3PO (0 and 10 µM) and cisplatin (0 and 20 µM) for 48 h, cell apoptosis was detected by Annexin V- FLUOS staining. **(D)** Protein expression of PARP, cleaved PARP, c-IAP1, c-IAP2 and survivin in A2780CP cells treated with 3PO (0 and 10 µM) and cisplatin (0 and 20 µM) for 48 h, determined by immunoblotting. **(E)** Bar chart of BIRC2, BIRC3, BIRC5 and CD70 genes mRNA expression in A2780CP cells treated with 3PO (0 and 10 µM) and cisplatin (0 and 20 µM) for 48 h, determined by the human apoptosis RT^2^ profiler PCR array. (n = 3; *p < 0.05; **p < 0.01).

To study the effect of PFKFB3 on chemoresistance of ovarian cancer, we first detected PFKFB3 expression in A2780S and A2780CP cells after cisplatin treatment (0, 2.5, 5 and 10 µM) for 48 h. Our findings of decreased PFKFB3 protein expression in A2780S cells but not in A2780CP cells after cisplatin treatment (**Figure 2A**), suggesting a differential regulation of PFKFB3 expression between sensitive and resistant ovarian cancer cells by cisplatin treatment. We further detected that treatment of chemoresistant A2780CP cells with 3-(3-pyridinyl)-1-(4-pyridinyl)-2-propen-1-one (3PO), the first compound for PFKFB3 inhibition (20), could decrease lactate production (**Figure 2B**). Then, we investigated if 3PO would abolish drug resistance of A2780CP by treating cells with 3PO and cisplatin for 48 h. Cisplatin (20 µM) treatment without 3PO caused 14.8% apoptotic cells, whereas cisplatin (20 µM) and 3PO (10 µM) combined treatment caused 31.55% (**Figure 2C**), suggesting that 3PO enhanced the effect of cisplatin in treating cancer cells. Moreover, increased cleavage of PARP was observed in cisplatin and 3PO combined treatment (**Figure 2D**).

It is generally believed that chemoresistance is in part contributed by the inability of cells to undergo apoptosis. To determine the apoptotic pathways by which 3PO attenuates the chemoresistant phenotype of cancer cells, the expression of 84 apoptosis-related genes in cisplatin and 3PO treatment was evaluated using the human apoptosis RT^2^ profiler PCR array (**Figure 2E**). We found cisplatin induced mRNA expression of the inhibitors of apoptosis (IAP) proteins including BIRC2 (c-IAP1), BIRC3 (c-IAP2) and BIRC5 (survivin) and an immune modulator CD70 (TNFSF7) in A2780CP cells. We further detected same pattern of c-IAP1, c-IAP2 and survivin in protein levels (**Figure 2D**), suggesting 3PO could sensitize ovarian cancer cells *via* altering expression of c-IAP1, c-IAP2, survivin and CD70.

### PFKFB3 Shifts CSCs Metabolism in Ovarian Cancer

**Figure 3:**
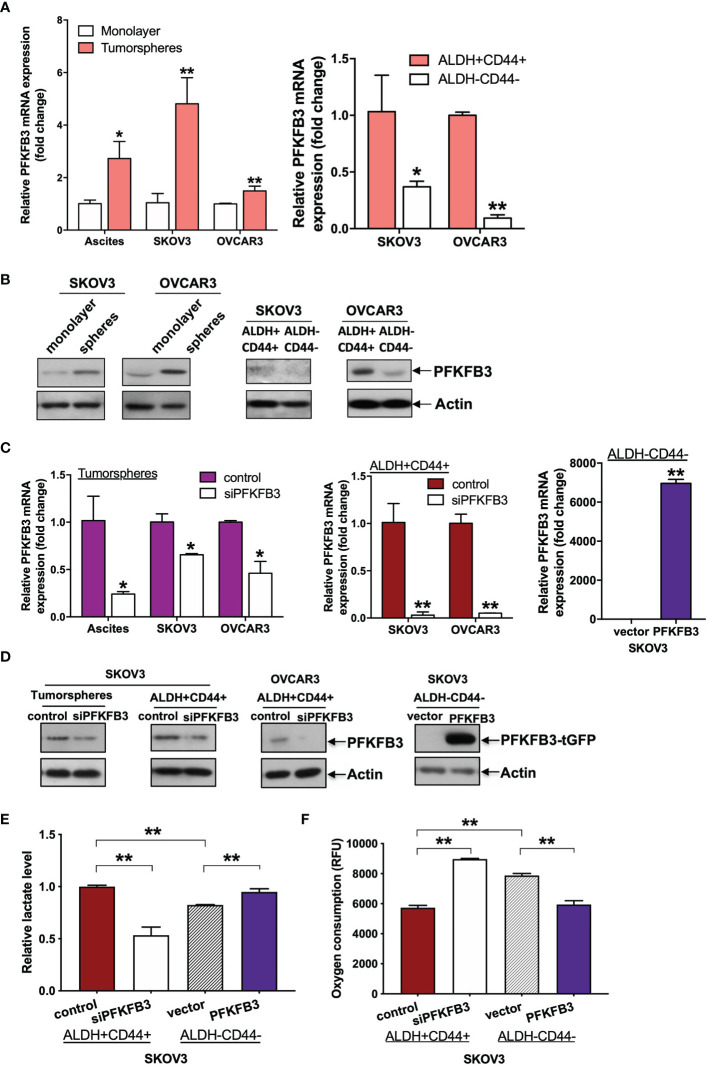
PFKFB3 shifts CSC metabolism in ovarian cancer. **(A)** Relative mRNA expression of PFKFB3 in tumorsphere/monolayer and ALDH+CD44+/ALDH-CD44- cells *via* qPCR. **(B)** Protein expression of PFKFB3 in tumorsphere/monolayer and ALDH+CD44+/ALDH-CD44- cells, determined *via* immunoblotting; Relative PFKFB3 **(C)** mRNA and **(D)** protein expression in tumorspheres and ALDH+CD44+ cells transfected with control siRNA or siPFKFB3 duplexes and PFKFB3-overexpressing ALDH-CD44- cells, determined by qPCR and immunoblotting, respectively. **(E)** Relative lactate production and **(F)** oxygen consumption rate of PFKFB3-suppressing ALDH+CD44+ cells and PFKFB3-overexpressing ALDH-CD44- cells after 24 h incubation. (n = 3; *p < 0.05, **p < 0.01).

Besides chemoresistance, stemness also play vital roles in the progression of ovarian cancer. In the present study, we applied two methods for enriching CSCs from ascites of ovarian cancer. One method involved maintaining tumorspheres in ultra-low attachment plates with serum-free medium. The other method involved sorting the top 5% brightest staining cells with stem cell markers (ALDH and CD44) by FACS, on the basis of our recent findings that ALDH+CD44+ cells isolated from ascites-derived tumor cells show enhanced CSC properties (16). Compared with that in monolayer or ALDH-CD44- cancer cells, significantly higher PFKFB3 mRNA expression was clearly detected in tumorspheres derived from ascites and ascites-derived cell lines (SKOV3 and OVCAR3) or ALDH+CD44+ cells sorted from SKOV3 and OVCAR3 cells (**Figure 3A**). Moreover, greater protein expression of PFKFB3 was found in tumorspheres or ALDH+CD44+ cells than monolayer/ALDH-CD44- SKOV3 cells (**Figure 3B**). There results indicated high expression of PFKFB3 in CSC-enriched subsets derived from ascites in ovarian cancer.

We have recently found that tumorspheres/ALDH+CD44+ cells favor glycolysis more than OXPHOS (16). To study the role of PFKFB3 in altered CSC metabolism in ovarian cancer, we performed metabolic assays using tumorspheres/ALDH+CD44+ cells transfected with siPFKFB3 duplexes and ALDH-CD44- cells transfected with PFKFB3. First, qPCR (**Figure 3C**) and western blot (**Figure 3D**) analyses were performed to validate the transfection efficiency. We then found that PFKFB3 knockdown led to decreased lactate production (**Figure 3E**) and an increased oxygen consumption rate (**Figure 3F**) in ALDH+CD44+ cells, whereas PFKFB3 overexpression resulted in increased lactate production and decreased the oxygen consumption rate in ALDH-CD44- cells. These data suggested that PFKFB3 could increase glycolytic influx and decrease OXPHOS activity in ALDH+CD44+ cells.

### PFKFB3 Promotes Stemness in Ovarian Cancer, Probably Through the NF-κB Signaling Pathway

**Figure 4:**
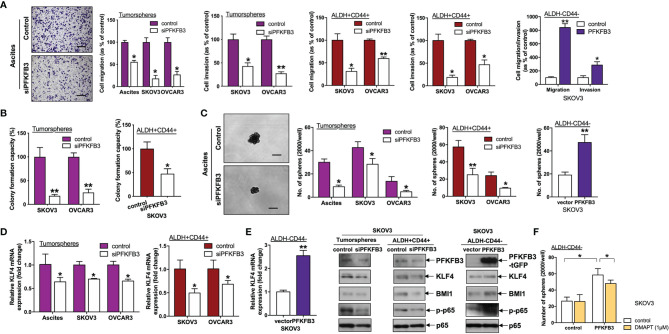
PFKFB3 promotes stemness in ovarian cancer. **(A)** Transwell migration/invasion assays of PFKFB3-suppressing tumorspheres and ALDH+CD44+ cells and PFKFB3-overexpressing ALDH-CD44- cells. Representative images of migrating tumorspheres with transient knockdown of PFKFB3 (left; scale bar, 100 μm). **(B)** Clonogenic assay of tumorspheres and ALDH+CD44+ cells transfected with control and siPFKFB3 duplexes cells. **(C)** Sphere formation assay of PFKFB3-suppressing tumorspheres and ALDH+CD44+ cells and PFKFB3-overexpressing ALDH-CD44- cells. Representative images of sphere-forming cells with transient knockdown of PFKFB3 (left; scale bar, 100 μm). **(D)** Relative KLF4 mRNA expression of PFKFB3-suppressing tumorspheres and ALDH+CD44+ cells and PFKFB3-overexpressing ALDH-CD44- cells, determined by qPCR. **(E)** Protein expression of PFKFB3, KLF4, BMI1, p-p65 and p65 in PFKFB3-knockdown tumorspheres (left) ALDH+CD44+ (middle) SKOV3 cells and PFKFB3-overexpressing ALDH-CD44- (right) SKOV3 cells, determined by immunoblotting. **(F)** After DMAPT (1 μM) treatment of PFKFB3-overexpressing ALDH-CD44- cells for 48 h, sphere formation assays were conducted and analyzed. (n = 3; *p < 0.05, **p < 0.01).

Next, we studied the role of PFKFB3 in regulating CSCs in ovarian cancer. We found that siPFKFB3-transfected tumorspheres/ALDH+CD44+ cells exhibited significantly less migration/invasion (**Figure 4A**) and formation of colonies (**Figure 4B**) and spheres (**Figure 4C**) than groups transfected with control siRNA. Moreover, transient overexpression of PFKFB3 in ALDH-CD44- cells led to the opposite effects (**Figures 4A–C**). GEPIA was used to predict the positive correlation between PFKFB3 and the stemness genes, NANOG, OCT4, SOX2, KLF4 and BMI1. A significant albeit very weak correlation was detected between high expression of PFKFB3 and upregulation of KLF4 and BMI1 (**Supplementary Figure 2**), suggesting KLF4 and BMI1 as the potential downstream targets of PFKFB3 on CSC properties in ovarian cancer. No significant correlation was found between PFKFB3 and NANOG, OCT4 and SOX2 (data not shown). We further found that decreased KLF4 and BMI1 mRNA and/or protein expression was observed in PFKFB3 suppressed tumorspheres/ALDH+CD44+ cells, whereas increased KLF4 and BMI1 expression was found in PFKFB3 overexpressing ALDH-CD44- cells (**Figures 4D, E**).

Numerous pathways are involved in CSC regulation in ovarian cancer, including NF-κB pathways (20). Phosphorylation of p65 at S536 (p-RelA) is one of the canonical NF-κB activation indicators (21). We further found that p-p65 was decreased in ALDH+CD44+ SKOV3 cells transfected with siPFKFB3 as compared with control cells (**Figure 4E**). Conversely, PFKFB3-overexpressing ALDH-CD44- SKOV3 cells had higher expression of p-p65 (**Figure 4E**). In addition, to assess the effect of NF-κB on stemness mediated by PFKFB3, we treated both ALDH-CD44- SKOV3 cells with ectopic PFPFB3 expression and control cells with the NF-κB inhibitor, dimethylaminoparthenolide (DMAPT), for 48 h. DMAPT inhibited the increased sphere formation capacity induced by the PFKFB3 plasmid in ALDH-CD44 cells but had no effects on the ALDH-CD44- cells transfected with the control plasmid (**Figure 4F**). Together, our results indicated that PFKFB3 regulates CSC properties in ovarian cancer, possibly through the NF-κB signaling pathway.

### PFK158 Suppresses Stemness in Ovarian Cancer

**Figure 5:**
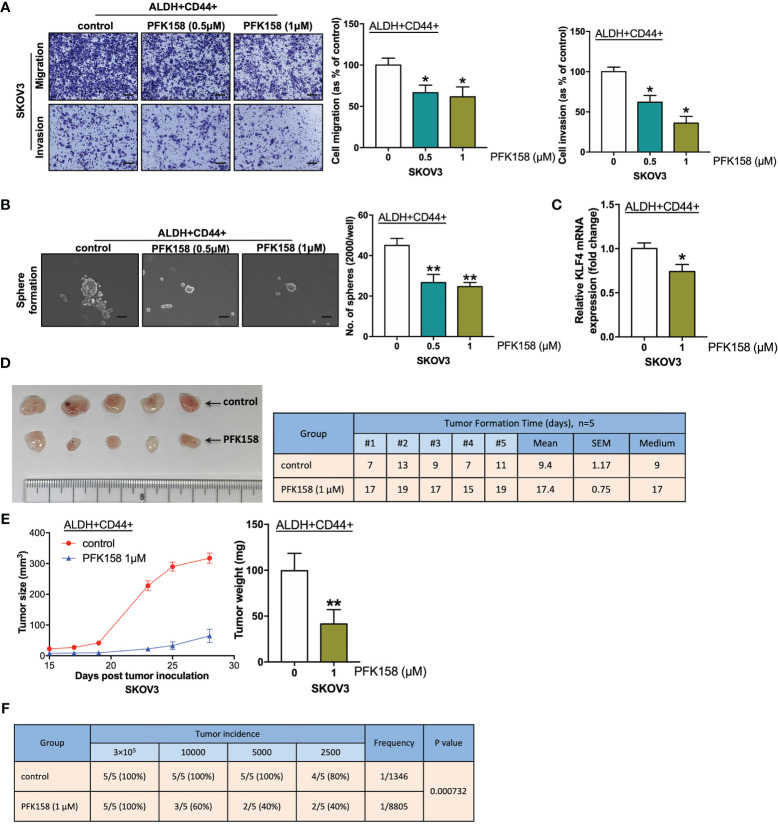
PFK158 suppresses stemness in ovarian cancer. **(A)** Transwell migration and invasion (scale bar, 100 μM) and **(B)** sphere formation (scale bar, 100 μM) of ALDH+ CD44+ SKOV3 cells pretreated with control and PFK158 (0.5 and 1.0 μM) for 48 h. **(C)** Relative KLF4 mRNA expression of ALDH+CD44+ SKOV3 cells treated with 1.0 μM PFK158, determined by qPCR. (n=3; *p < 0.05, **p < 0.01). **(D)** After inoculation of 3×10^5^ ALDH+CD44+ SKOV3 cells pretreated with control (n = 5) and PFK158 (1.0μM, n = 5) into the right/left flanks of mice, images of dissected tumors and tumor formation days were recorded. **(E)** Line chart showing the tumor growth and bar chart showing the weights of tumors derived from the mice injected with 3×10^5^ control and PFK158-pretreated ALDH+CD44+ SKOV3 cells. **(F)** Tumor incidence and estimated frequency from *in vivo* limiting dilution assays with injection of a limiting dilution (3×10^5^, 10×10^3^, 5×10^3^, or 2.5×10^3^ cells) of control and PFK158-pretreated ALDH+CD44+ SKOV3 cells into the flanks of mice.

PFK158 is a PFKFB3 inhibitor that mimics genetic inhibition of PFKFB3 in malignant pleural mesothelioma (22). Here, ALDH+ CD44+ cells were treated with PFK158 and examined with functional assays to evaluate the effects of PFK158 on the CSC subpopulation in SKOV3 cells. PFKFB3 inhibited migration/invasion (**Figure 5A**) and sphere formation (**Figure 5B**) capacity in ALDH+CD44+ SKOV3 cells. Moreover, KLF4 mRNA expression decreased in ALDH+CD44+ cells suppressed by PFK158 (**Figure 5C**). To determine the effect of PFK158 on tumorigenesis *in vivo*, we injected control or PFK158-pretreated ALDH+CD44+ SKOV3 cells into the flanks of nude mice. The number of days of tumor formation and tumor sizes were recorded. After 28 days, tumors were dissected and weighed. PFK158-pretreated ALDH+CD44+ cells formed smaller tumors, and the tumor formation time was prolonged from an average of 9.4 days to 17.4 days (**Figure 5D**). Moreover, tumor size and weight were significantly lower in the PFK158-pretreated group than the control group (**Figure 5E**). We used *in vivo* limiting dilution assays with a serial dilution of PFK158/control-pretreated ALDH+CD44+ SKOV3 cells to determine the effect of PFK158 on tumor-initiating properties. Tumor-incidence declined from 100% to 60% in the 10**^4^**-cell group, 100% to 40% in the 5000-cell group, and 80% to 40% in the 2500-cell group with PFK158-pretreated ALDH+CD44+ SKOV3 cells (**Figure 5F**). These findings suggested that PFK158 suppresses stemness in ovarian cancer.

## Discussion

We found through IHC analysis that PFKFB3 was markedly higher in ovarian cancer tissues than normal ovary tissues, a finding consistent with Oncomine analysis results. PFKFB3 was also associated with tumor stage, grade and serous subtype. Moreover, higher expression of PFKFB3 decreased the survival rate and correlated with metastasis, thus, suggesting that PFKFB3 may be a novel prognostic marker for ovarian cancer. Concordantly, PFKFB3 is correlated with the aggressive features of breast cancer and indicates poorer prognosis in breast cancer and hepatocellular carcinoma (15, 23). In gastric cancer, PFKFB3 is preferentially expressed in tumors with lymph node metastasis (24). In the present study, we demonstrate for the first time to the best of our knowledge that PFKFB3 is preferentially expressed in metastatic foci and ascites in ovarian cancer, suggesting that PFKFB3 could be associated with metastasis in ovarian cancer.

Here, blocking PFKFB3 by 3PO could inhibit lactate production and enhance the sensitivity of A2780CP cells to cisplatin. Further, we noticed cisplatin induced IAPs mRNA/protein expression including c-IAP1, c-IAP2 and survivin and an immune modulator CD70 in A2780CP cells, whereas 3PO could abolish their mRNA/protein induction, suggesting 3PO could sensitize ovarian cancer cells to cisplatin possibly through the modulation of c-IAP1, c-IAP2, survivin and CD70 mRNA expression. Increase expression of c-IAP1, c-IAP2 and survivin after cisplatin treatment in resistant cells has been documented (25, 26), suggesting their roles on cisplatin resistance (27, 28). Blocking survivin through knockout or inhibitor could suppress metastasis and chemoresistance (29). Also, the association between CD70 and clinical cisplatin resistance and poor prognosis of advanced ovarian cancer has been recorded (30, 31), yet the precise mechanism remains to be elucidated. These results strongly supported PFKFB3 as a mediator of cisplatin resistance in ovarian cancer.

Recent studies have suggested that CSCs show an altered energy balance, with glycolysis being the major metabolic phenotype in cancers of the liver, breast and colon (6). In ovarian cancer, anaerobic glycolysis is enhanced in tumorspheres derived from cancer cells (3). In our recent study, we further discovered that tumorspheres/ALDH+CD44+ cells favor glycolysis more than OXPHOS (16). Here, we found that PFKFB3, another key enzyme in promoting glycolysis, was up-regulated in ascites-derived cancer cells. Furthermore, tumorspheres and the ALDH+CD44+ cell subset originating from ascites had greater expression of PFKFB3 than differentiated cancer cells. These findings further support that CSC-enriched subpopulations such as in ALDH+CD44+ cell subset derived from ascites favor glycolysis over OXPHOS.

Functionally, knockdown of PFKFB3 results in a reduced migration rate of gastric cancer cells (24), a result consistent with our finding that manipulation of PFKFB3 in CSC-enriched/differentiated subsets affected the migration/invasion capacity of ovarian cancer *in vitro*. Silencing of PFKFB3 decreases glucose consumption and inhibits DNA repair; thus, suppressing tumor growth in hepatocellular carcinoma (23). Our study not only found upregulated PFKFB3 in CSC-enriched ovarian cancer cells, but also revealed that PFKFB3 promoted clonogenicity and sphere-formation, and induced KLF4 and BMI1 expression in ovarian cancer cells.

In terms of downstream signaling, PFKFB3 binds and activates CDK4, a kinase controlling the G1/S transition, thus, promoting cell cycle progression (32). Moreover, silencing of PFKFB3 deactivates CDK1 and stabilizes p27 protein expression, thereby leading to G1/S arrest and enhanced apoptosis in HeLa cells (14). A recent study revealed that up-regulation of PFKFB3 promoted immune evasion and tumorigenesis by inducing PD-L1 expression through NF-κB activation in hepatocellular carcinoma (33). NF-κB pathways have also been suggested to be involved in CSC regulation of ovarian cancer (20). Here, we observed that silencing of PFKFB3 decreased NF-κB activation, whereas ectopic expression of PFKFB3 up-regulated NF-κB activation. Not all functional assays (overexpression studies) were performed in all cell lines and SKOV3 may not be a representative of the most common high-grade serous ovarian cancer, the use of other ovarian cancer cell lines for performing function assays after overexpression of PFKFB3 would be examined in further studies.

We further evaluated the therapeutic potential of targeting PFKFB3. The compound 3PO has been identified to be an inhibitor of PFKFB3 (19) and has been reported to attenuate glycolytic activity and attenuate tumor growth in bladder, liver, breast (11) and colon cancers (34). Blockade of PFKFB3 by 3PO in mutant JAK2-driven myeloproliferative neoplasms alters redox homeostasis through inhibiting glycolysis, thus, resulting in accumulation of reactive oxygen species and increased apoptosis (35). In ovarian cancer, 3PO blocks glycolysis, thus, causing increased extracellular glucose and impeded lactate secretion, induces apoptosis (36) and enhances the sensitivity of tumor cells to platinum therapy (37). However, 3PO has not been applied clinically because of its poor water solubility (11). Another potent selective PFKFB3 inhibitor, PFK158, is the first PFKFB3 inhibitor being approved by the U.S. Food and Drug Administrator (FDA) to undergo clinical trial in patients with various malignancies including ovarian, prostate, lung, melanoma, breast and pancreatic cancers (http://www.advancedcancertherapeutics.com). In combination with immune checkpoint inhibitors, PFK158 has been found to decrease tumor growth in a preclinical mouse model in melanoma (38). A previous study has suggested that PFK158 combined with chemotherapy increases apoptosis in melanoma cells (39). As a small molecular antagonist of PFKFB3, PFK158 can mimic genetic inhibition of PFKFB3 (thus inhibiting glycolytic activity), arrest tumor cells in G0/G1 phase, and promote cell death as well as enhance chemotherapy in malignant pleural mesothelioma (22). Recently, PFK158 has been reported to inhibit glucose metabolism and enhance the sensitivity of tumor cells to carboplatin in ovarian and cervical cancer cells (40). Here, we extended the function of PFK158 to CSC-enriched subpopulations. Our study showed that PFK158 attenuates sphere formation, migration/invasion and KLF4 mRNA expression, and enhances the sensitivity of ALDH+CD44+ cancer cells. Moreover, PFK158 impedes tumorigenesis and tumor-initiation *in vivo*.

## Conclusions

In conclusion, we demonstrated that overexpression of PFKFB3 in ovarian cancer cells, especially in the CSC subpopulation, correlates with metastasis and patient survival. PFKFB3 could induce lactate production, chemoresistance, CSC properties and tumor growth in ovarian cancer through inhibition of apoptosis and NF-κB signaling pathway. Moreover, our evidence suggests the therapeutic potential of PFK158 as an inhibitor of PFKFB3 in targeting ovarian cancer.

## Data Availability Statement

The original contributions presented in the study are included in the article/**Supplementary Material**. Further inquiries can be directed to the corresponding author.

## Ethics Statement

The use of patient samples was approved by the Institution Ethical Review Board of the University of Hong Kong (UW 16-107). The patients/participants provided their written informed consent to participate in this study. This study was approved by the Committee of the Use of Live Animals in Teaching and Research (CULATR) under an approved license (No. 4598-18).

## Author Contributions

Y-xJ, MS, and KC conceived the project. Y-xJ, MS, and J-jJ performed the experiments. TL, DC, and AC contributed new reagents/analytic tools. Y-xJ, MS, HN, and KC analysed the data. Y-xJ and MS wrote the manuscript. MS and KC supervised the study. All authors provided critical revision and approved the final manuscript.

## Funding

The work was jointly funded by the University of Hong Kong (201411159066) and by the Hong Kong Research Grants Council General Research Fund (HKU 17101414), and the Research Fund from the Department of Obstetrics and Gynaecology.

## Conflict of Interest

The authors declare that the research was conducted in the absence of any commercial or financial relationships that could be construed as a potential conflict of interest.

## Publisher’s Note

All claims expressed in this article are solely those of the authors and do not necessarily represent those of their affiliated organizations, or those of the publisher, the editors and the reviewers. Any product that may be evaluated in this article, or claim that may be made by its manufacturer, is not guaranteed or endorsed by the publisher.
