# Supplementary material for: PFKFB3 Regulates Chemoresistance, Metastasis and Stemness via IAP Proteins and the NF-κB Signaling Pathway in Ovarian Cancer
Source: Front Oncol. 2022 Jan 28;12:748403. doi: 10.3389/fonc.2022.748403 (PMC8837381; doi:10.3389/fonc.2022.748403)
Supplement: Supplementary file 1 [file DataSheet_1.docx]

Supplementary Material

## Supplementary Figures


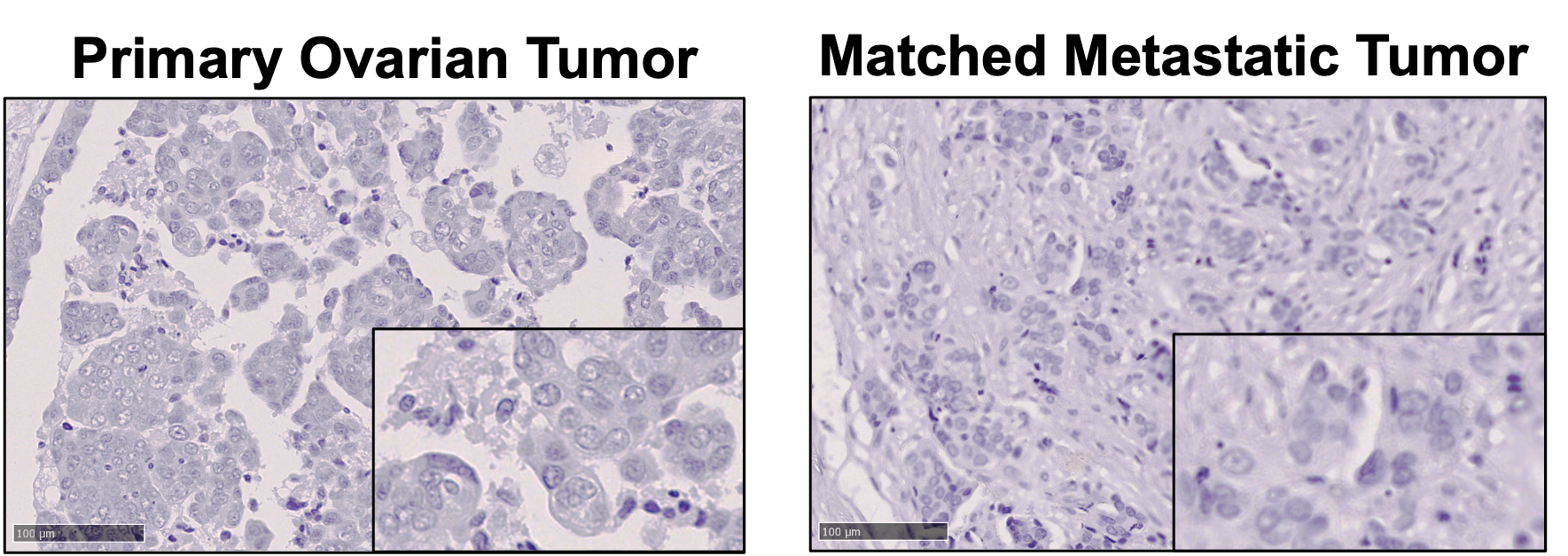


**Supplementary Figure 1.** Images of IHC staining for negative control with primary antibody replaced with PBS in ovarian primary tumor (i) and its matched metastatic foci (ii; scale bar, 100 μm). The insets highlight regions with higher magnification.

**Supplementary Figure2.** Correlation coefficients of PFKFB3 with KLF4 and BMI1 in ovarian cancer data retrieved from the TGCA database. GEPIA was used to plot the expression Z-scores of PFKFB3 and KLF4/BMI1.

## Supplementary Tables

| **Characteristics** | **Case (n)** |
| --- | --- |
| **Stage (FIGO)** |  |
| 1 | 53 |
| 2 | 19 |
| 3 | 25 |
| **Histological grade (FIGO)** |  |
| I-II | 31 |
| II-III | 66 |
| **Histology** |  |
| Serous | 50 |
| Mucinous | 18 |
| Endometrioid | 28 |
| Clear Cell | 2 |

**Supplementary Table S1.** Clinicopathological characteristics of 97 samples in the tissue microarray (TMA, OVC1021; Biomax).
